# Personality and nonjudging make you happier: Contribution of the Five-Factor Model, mindfulness facets and a mindfulness intervention to subjective well-being

**DOI:** 10.1371/journal.pone.0228655

**Published:** 2020-02-04

**Authors:** Generós Ortet, Daniel Pinazo, Diane Walker, Sígrid Gallego, Laura Mezquita, Manuel I. Ibáñez

**Affiliations:** 1 Department of Basic and Clinical Psychology and Psychobiology, Universitat Jaume I,Castelló, Spain; 2 Centre for Biomedical Research Network on Mental Health (CIBERSAM), Instituto de Salud Carlos III, Madrid, Spain; 3 Department of Developmental, Educational and Social Psychology and Methodology, Universitat Jaume I,Castelló, Spain; University of Lleida, SPAIN

## Abstract

Mindful individuals are able to acknowledge mind wandering and live in the present moment in a nonjudgmental way. Previous studies have found that both mind wandering and mindfulness are associated with subjective well-being. However, the main predictor of happiness is personality; more specifically, happier people are emotionally stable and extraverted. The present study aimed to explore the contribution of the five factors of personality, dispositional mindfulness facets and a mindfulness intervention to happiness. A sample of 372 university students was assessed with the NEO-Five Factor Inventory, and another sample of 217 community adults answered the Big Five Personality Trait Short Questionnaire. Both samples, 589 participants in all, completed the Five Facet Mindfulness Questionnaire and the Subjective Happiness Scale. Furthermore, 55 participants from the general population sample took a 6-week training course in meditation and developing mindfulness. The regression analyses showed that emotional stability and extraversion traits were the strongest predictors of subjective well-being. Nonetheless, the nonjudging facet, which is nonevaluative/acceptance awareness of thoughts and feelings, still remained a significant predictor of happiness when personality was accounted for. Finally, mindfulness training did not increase subjective well-being. Being nonjudgmental of one’s inner thoughts, feelings and sensations contributes to happiness even when personality is taken into account. Accordingly, it seems reasonable that mindfulness training that intends to improve subjective well-being should focus on noticing thoughts without judging them.

## Introduction

Mindfulness has received considerable attention in the last few years in both applied and basic research psychology [1]. For instance, mindfulness-based psychological interventions [2,3] are often used to treat psychological disorders (e.g., mood and anxiety disorders, addictions; [4]) and health conditions (e.g., chronic pain, cancer; [5]). In basic research, special interest is shown in dispositional or trait mindfulness and its measurement [6]. Although not undisputed consensus about the definition of mindfulness has been reached [1], being mindful defines a nonjudgmental, accepting and nonreactive attention or awareness of the present moment [6–8]. It seems clear that the experience of states of mindfulness varies, on average, from person to person, which suggests the existence of a dispositional tendency toward mindfulness or stable individual differences in mindfulness [9]. Accordingly, dispositional mindfulness is conceptualized as a personality-like trait that refers to the tendency to be mindful in everyday life [7,9]. Mindfulness is predominantly measured using self-report questionnaires devised to assess this general disposition. Of these scales, the Five Facet Mindfulness Questionnaire (FFMQ; [10,11]) is one of the most widely used. The FFMQ assesses five facets: observing, describing, acting with awareness, nonjudging, and nonreactivity. Moreover, dispositional mindfulness seems susceptible to change with practice and training, according to a recent systematic review and meta-analysis [8]. Several former studies have found that greater predisposition to be mindful helps the person to control automatic thoughts, prevents unhealthy lifestyles, and improves self-regulation behaviors and interpersonal relationship quality [7]. The results of a meta-analysis have indicated that dispositional mindfulness correlates with confidence, mental health, emotional regulation and life and job satisfaction, and is negatively associated with perceived life stress, anxiety and depression [9].

Mindful individuals are able to manage mind wandering by coming back to the present moment [12]. Previous studies have found that both mind wandering and mindfulness (state and dispositional) are associated with subjective well-being [7,13–16]. Mindfulness may enhance well-being because awareness facilitates paying attention to the prompts that arise from basic psychological needs, which makes one more likely to regulate behavior so that it fulfills such needs [17]. The practice of mindfulness is also believed to improve self-observation, which promotes the recognition of internal states, understanding the consequences of one’s actions, and improving the ability to use appropriate coping skills [12,18,19]. Evidence for the benefits of mindful awareness and attention have come from research which has shown that mindfulness training is related to positive psychological outcomes in general [2,20,21], and to happiness in particular [15,16,22]. The results on the relationships of the particular aspects of mindfulness and well-being are mixed. Previous studies have shown that all the FFMQ facets except observing predict psychological well-being [10], but others have found that none of the mindfulness aspects predict happiness [23]. More recent findings indicate that describing and nonreactivity are the main aspects associated with either subjective well-being [24] or life satisfaction [25]. Thus, it seems clear that more research is needed to clarify which mindfulness aspects are associated with happiness. Furthermore, most previous studies have not considered personality traits when exploring the association between trait mindfulness and subjective well-being. Notwithstanding, research into the psychological factors that influence happiness shows that the main predictor of happiness is personality [26,27].

Most research on personality traits has been performed according to the Five-Factor Model (FFM). The FFM has become a model with wide consensus among personality psychologists and offers a useful descriptive taxonomy of personality traits [28,29]. The FFM (a.k.a. Big Five), proposes the broad traits of neuroticism, extraversion, openness to experience, agreeableness and conscientiousness. Neuroticism reflects individual differences in the predisposition to experience negative emotions (e.g., anxiety, depressive mood, fear, irritability or low self-esteem) frequently and intensively; extraversion refers to individual differences in warmth, sociability, dominance, activity, excitement-seeking, and the tendency to experience positive emotions (e.g., joy, cheerfulness or enthusiasm) frequently and intensively; openness reveals individual differences in intellectual curiosity, appreciation of artistic beauty, imagination, consideration for inner feelings, preference for variety, and tolerance of diversity; agreeableness represents individual differences in trust, compliance, tender-mindedness, cooperation, modesty, and altruism; conscientiousness implies individual differences in being disciplined, organized, cautious, achievement-striving, and dutiful [30]. The study of the relationship between personality and subjective well-being has shown that happier people are extraverted and emotionally stable [26,27]. Two of the main components of happiness are positive and negative affect, which are associated with extraversion and neuroticism, respectively [31]. In relation to the associations between mindfulness and the FFM personality traits, it has been found that (low) neuroticism, conscientiousness and openness were the strongest predictors of dispositional mindfulness [6]. Accordingly, mindful individuals tend to have a low susceptibility to psychological distress, high impulse control, are not easily distracted, pay attention, and are curious and receptive. The other two personality dimensions, agreeableness and extraversion, also show moderate relationships with mindfulness [32]. Regarding mindfulness facets, previous results (e.g., [33]) have indicated that each dispositional mindfulness facet, except for observing, is associated with low neuroticism. Furthermore, the describing, acting with awareness, and nonjudging facets characterize conscientious, agreeable and extraverted individuals. Openness seems to be more closely linked to the mindful tendencies of observing and describing experience.

The fact that basic personality dimensions become increasingly stable throughout adulthood [34] indicates that they will better resist modification. Conversely, dispositional mindfulness would be more malleable than basic personality traits [24]. For instance, mindfulness training seems to increase dispositional mindfulness and subjective well-being [35–37]. Accordingly, and by contemplating the above-mentioned relationships between the FFM and mindfulness, dispositional mindfulness may be considered a proximal consequence of basic personality traits, which is an ongoing matter of debate [38]. According to different studies, as dispositional mindfulness and mindfulness training are related to happiness, they may contribute to subjective well-being over and above the FFM broad dimensions. However, as mentioned before, very little is known about the predictive role of dispositional mindfulness in happiness controlling for the effects of the five factors of personality. For instance, [24] studied the associations linking neuroticism, trait mindfulness and psychological well-being, but did not consider the other four personality factors.

The aim of the present study was to examine the influence of the five broad domains of personality, the five facets of dispositional mindfulness and a mindfulness intervention to subjective well-being. According to the previous findings on the associations between mindfulness and happiness, it was hypothesized that the five facets of dispositional mindfulness would predict subjective well-being beyond the five factors of personality. As different studies have shown, mindfulness practice was also expected to increase both dispositional mindfulness (the total score and the five facets) and happiness.

## Materials and methods

### Participants and procedures

Sample 1 consisted of 372 university students (81.5% females), whose mean age was 20.71 years (*SD* = 4.20; age range 18–53 years). The participants came from different parts of Spain, but most lived in the Valencian Community (east Spain). They answered the questionnaires over the internet. They filled out the scales as a response to an advertisement displayed in the virtual classrooms at the authors’ university. The participants gave their consent to participate in the research by clicking the protection data and consent information button in the first page of the internet survey.

Sample 2 comprised 217 participants (82.9% females) with a mean age of 27.20 years (*SD* = 11.20; age range 18–77 years), who were recruited from the general population. Overall, they had high levels of education with 33.64% holding a university degree, 60.83% having finished non-compulsory secondary education, and only 5.53% had compulsory studies. They all lived in the Valencian Community. The participants were contacted by several means, including social media platforms, which offered them the chance to participate in a study that involved answering different measures in the paper-and-pencil format, and the possibility of taking a free 6-week intervention in meditation and mindfulness at the authors’ university. They had to attend a talk in one of the classrooms with a scheduled timetable, and complete the questionnaires. They gave their consent orally before starting to answer the measures, which was recorded by two of the authors by writing down the participant’s name. Written consent was not obtained because this particular procedure had been approved by the Deontological Committee at the authors’ university.

The participants from Sample 2, who enrolled in the 6-week training course in meditation and developing mindfulness, formed Subsample 2A. It comprised 55 participants (81.8% females), whose mean age was 32.27 years (*SD* = 11.54; age range 19–65 years). One experienced instructor (a co-author) ran all the training courses following the same methodology for both groups: one with 25 participants and the other with 30. He is a senior lecturer in psychology and has completed the mindfulness-based stress reduction (MBSR) course taught by a fully certified MBSR instructor. He has attended four 1-week long meditation retreats in the last 4 years. He has been a mindfulness trainer at the authors’ university since 2010. Since 2017, he has begun to work as a management consultant in mindfulness. All the applicants were informed that, if they agreed to take part in the course, they would consent to participate in research into the effects of meditation on quality of life. They were also directly asked if they were receiving any intervention or treatment for psychological disorders at the time. None of the 55 participants received treatment, so they all were accepted for this mindfulness intervention adapted to a nonclinical population. However, no screening tool for psychological disorders was used.

The two consent procedures, i.e., a) clicking a button in the internet survey, and b) orally to two of the authors both before administering the first paper-and-pencil test and before starting the intervention course, were approved by the Deontological Committee at the authors’ university.

The five broad personality traits were measured using the NEO-Five Factor Inventory [39] in Sample 1 and the Big Five Personality Trait Short Questionnaire [40] in Sample 2. The other two scales employed in the present study, the Five Facet Mindfulness Questionnaire-15 [41] that assesses dispositional mindfulness, and the Subjective Happiness Scale [42] that measures happiness, were the same for all the participants. Finally, Subsample 2A once again answered the three questionnaires 2 months later, when mindfulness training had ended. Two different samples were used in order to test, in independent samples of participants, if dispositional mindfulness could explain happiness beyond personality traits. However, the questionnaires used to measure the five personality traits were different in each sample for practical and research reasons. Thus the two samples formed part of a broader study on personality and mindfulness, including the adaptation of a noncommercial personality questionnaire, the Big Five Personality Trait Short Questionnaire. As different assessment tools for personality were used, not replicating the results in the two samples was more probable, which more strongly supported the hypotheses if the predictions were confirmed in both samples.

### Mindfulness training

The mindfulness training sessions were adapted for a nontherapeutic population. Drawing on the structure of MBSR, the adaptation followed the theoretical perspective of the relationship level principles described in systemic communication theory (e.g., [43]). Intervention took place over six 2-hour sessions designed to develop the participants’ ability to observe their internal language and its effect on their social interaction.

The participants learned how to relate to their present moment experience by tuning into thoughts, emotions and body sensations. They practiced metacognition exercises by paying attention in a sustained steady manner, moment to moment, and making no judgment. In order to develop this mindset, practice centered especially on observing thoughts. Learning involved knowing how to differentiate between the story attached to experience and the actual present moment one. This helped the participants to develop awareness of expectations, emotional intentions or interpretations that we might have in social interactions. These perceptions affect our mood and, in turn, the way in which we relate to others. This Metacognition-Based Mindfulness and Meditation Program is registered (doi:10.13140/RG.2.2.18407.19367) and freely available S1 Fig.

### Measures

#### *Five Facet Mindfulness Questionnaire-15* (FFMQ-15; [41,44])

The FFMQ-15 is a short version of the FFMQ [8]. The FFMQ is one of the most widely used scales for assessing dispositional mindfulness. The questionnaire comprises five factors: observing, describing, acting with awareness, nonjudging of inner experience, and nonreactivity to inner experience. Observing comprises noticing or attending to internal and external experiences, such as cognitions, emotions or sense perception. Describing refers to identifying internal experiences. Acting with awareness includes paying attention to one’s activities at the time, as opposed to doing so in an automatic pilot mode and paying attention elsewhere. Nonjudging refers to taking a nonevaluative/acceptance stance to thoughts and feelings. Nonreactivity is the tendency to allow thoughts and feelings to come and go, without getting carried away by them [11]. The FFMQ-15 includes 15 items that assess these five facets (three items per facet). Items are rated on a 5-point Likert-type scale ranging from 1 (never or very rarely true) to 5 (very often or always true). The 15 items from the Spanish adaptation of the scale were used [45]. Cronbach’s alphas for the total mindfulness score were .69 for Sample 1, .74 for Sample 2 and .80 for Subsample 2A. With facets, the alphas were: observing .66, .57 and .56; describing .80, .82 and .88; acting with awareness .66, .78 and .75; nonjudging .83, .82 and .70; and nonreactivity .57, .71 and .74 for Sample 1, Sample 2 and Subsample 2A respectively. These were lower than the internal consistencies of the long 39-item version [10,45]. However, the alphas in the current study were similar to those found in the original short 15-item scale [41]. Moreover, the long and short FFMQ versions seemed to be comparable [44].

#### *NEO-Five Factor Inventory* (NEO-FFI; [46])

The NEO-FFI is a 60-item inventory that assesses the five broad domains of personality: neuroticism, extraversion, openness to experience, agreeableness, and conscientiousness. The participants answer items on a 5-point Likert-type scale that range from 0 (strongly disagree) to 4 (strongly agree). The manual summarizes the reliability and validity data of the Spanish version of the instrument [39]. Alpha reliabilities for Sample 1 were: neuroticism .85, extraversion .83, openness .82, agreeableness .73, and conscientiousness .88.

#### *Big Five Personality Trait Short Questionnaire* (BFPTSQ; [30])

The BFPTSQ consists of 50 items that assess the FFM broad dimensions on a 5-point response scale (0 = *totally disagree*, 4 = *totally agree*): openness, extraversion, agreeableness, conscientiousness and emotional stability. [40] show the reliability and validity results of the Spanish version of the scale. Alpha reliabilities were: openness .83 and .82, extraversion .88 and .86, agreeableness .80 and .70, conscientiousness .86 and .86, and emotional stability .89 and .90 for Sample 2 and Subsample 2A respectively.

#### *Subjective Happiness Scale* (SHS; [42])

The SHS is a 4-item self-report measure of subjective well-being. Each item has a 7-point Likert scale response format. Two members of the authors’ research team, proficient in English and Spanish and with expertise in test adaptation, translated the items to Spanish. A back translation was carried out by an external qualified English language teacher. The evaluation of the back translation showed that the Spanish version of the SHS could be considered the equivalent to the original scale. The internal consistency coefficients for the samples were .88 for Sample 1, .81 for Sample 2, and .79 for Subsample 2A. Alphas were similar to those in the original scale [42].

### Data analysis

Pearson correlations were used to explore the relations among the five broad domains of personality, dispositional mindfulness (total score and five facets) and subjective well-being. Multiple linear regression analyses were conducted separately for the two samples to examine the amount of incremental contribution of the five facets of dispositional mindfulness over and above personality in predicting happiness using the SPSS 25. To this end, gender and age (step 1), as is usually performed in regression analysis, and the five personality dimensions (step 2), as the main predictors of happiness according to previous studies [26,27], were included as covariates. Hence their effects were controlled for. In step 3, the five facets of dispositional mindfulness were introduced into the regression analysis, which meant that five tests were done. The Benjamini and Hocberg procedure was followed to correct the *p*-values for multiple testing [47].

The mean scores were compared before and after the mindfulness intervention in Subsample 2A using the repeated measures ANCOVA. Cohen’s *d* and partial eta squared indices were calculated as measures of effect size when comparing the mean scores between the pre- and post-intervention times. Cohen’s *d* values of .20, .50, and .80 correspond to small, medium, and large effect sizes, respectively [48].

## Results

Table 1 shows the correlations between the five broad traits of personality and the total score, and the facets of dispositional mindfulness in both samples. All the personality dimensions correlated significantly with general mindfulness. Neuroticism was inversely related to all the facets, except for observing. Extraversion presented the highest associations with describing and nonjudging. Openness was related mainly to observing. Agreeableness had high associations with acting with awareness and nonjudging. Finally, conscientiousness correlated mainly with acting with awareness. In relation to the association between personality and happiness, as expected, emotional stability and extraversion presented the highest correlations in both samples (see Table 2). Dispositional mindfulness was also associated with subjective well-being, and nonjudging was the facet that explained more variance (around 23% and 18% in Samples 1 and 2, respectively; see Table 2).

**Table 1:** Correlations between personality and dispositional mindfulness.

	Mindfulness (total score)	Observing	Describing	Acting with awareness	Nonjudging	Nonreactivity
Sample 1 (*n* = 372)						
***NEO-FFI scales***						
**Neuroticism**	-.51***	.01	-.25***	-.33***	-.52***	-.33***
**Extraversion**	.31***	.11*	.32***	.09	.25***	.04
**Openness**	.23***	.43***	.18**	-.13*	-.03	.15**
**Agreeableness**	.18***	-.11*	.12*	.20***	.27***	.03
**Conscientiousness**	.27***	.01	.19***	.33***	.15**	.07
Sample 2 (*n* = 217)						
***BFPTSQ scales***						
**Emotional stability**	.52***	.02	.22**	.37***	.53***	.35***
**Extraversion**	.40***	.14*	.34***	.23**	.26***	.16*
**Openness**	.26***	.26***	.22**	-.07	.12	.22**
**Agreeableness**	.34***	-.10	.16*	.31***	.44***	.14*
**Conscientiousness**	.44***	.03	.27***	.54***	.33***	.11

*Note*. The BFPTSQ emotional stability scores indicate low neuroticism.

* *p* < .05.

** *p* < .01.

*** *p* < .001.

**Table 2:** Correlations of personality and dispositional mindfulness with subjective well-being.

		Subjective well-being	
***Personality scales***		Sample 1 (*n* = 372)	Sample 2 (*n* = 217)
	**Neuroticism/Emotional stability**	-.58***	.53***
	**Extraversion**	.59***	.48***
	**Openness**	.08	.16*
	**Agreeableness**	.35***	.44***
	**Conscientiousness**	.17**	.18**
***Mindfulness scales***			
	**Mindfulness (total score)**	.39***	.38***
	**Observing**	.04	.09
	**Describing**	.20***	.12
	**Acting with awareness**	.17**	.27***
	**Nonjudging**	.48***	.42***
	**Nonreactivity**	.16**	.18*

*Note*. The five factors of personality were assessed with the NEO-FFI in Sample 1 and with the BFPTSQ in Sample 2, which emotional stability scores indicating low neuroticism.

* *p* < .05.

** *p* < .01.

*** *p* < .001.

To examine the incremental contribution, and beyond personality, of dispositional mindfulness in predicting happiness, Table 3 presents the multiple linear regression analyses that predicted subjective well-being with the FFM personality traits in step 2 (after controlling for gender and age in step 1) and the five FFMQ scales in step 3. The results show that personality explained 51% in Sample 1 and 42% in Sample 2 of variance in the happiness scale. Thus the contribution of personality was practically the same in the two independent groups of participants, although personality traits were assessed using different inventories. The five dispositional mindfulness facets showed a significant incremental validity of 4% in Sample 1, but a nonsignificant 3% in Sample 2. Regarding beta weights, the nonjudging facet remained a significant predictor of subjective well-being in both samples. However, when the *p*-values were corrected for multiple testing, nonjudging was significant only in Sample 1. Compared to its nonsignificant bivariate correlation (*r* = .12 in Sample 2, see Table 2), the describing facet presented a significant inverse association with happiness (*β* = -.13) in Sample 2 after controlling for the other mindfulness facets. These contradictory results could reflect a multicollinearity problem and/or statistical suppressor situation [49]. It is noteworthy that the inverse analysis (incremental contribution of the FFM beyond the five dispositional mindfulness facets) yielded large effects (ΔR^2^ = .29 and .25 in Samples 1 and 2, respectively; *p* < .001).

**Table 3:** Multiple linear regression analyses predicting subjective well-being (SHS) with gender and age in step 1, the five factors of personality (NEO-FFI in Sample 1 and BFPTSQ in Sample 2) in step 2, and the five scales of mindfulness (FFMQ) in step 3.

		Subjective well-being			
**Predictor**		Sample 1 (*n* = 372)		Sample 2 (*n* = 217)	
		△R^2^	β	△R^2^	β
**Step 1**		.01		.01	
	**Gender**		.12*		
	**Age**		.02		
**Step 2**		.51***		.42***	
	**Neuroticism/Emotional stability**		-.41***		.39***
	**Extraversion**		.38***		.31***
	**Openness**		.01		.05
	**Agreeableness**		.13**		.16*
	**Conscientiousness**		.01		-.07
**Step 3**		.04***		.03	
	**Observing**		.03		.09
	**Describing**		-.07		-.13*
	**Acting with awareness**		-.06		.07
	**Nonjudging**		.22***		.14*
	**Nonreactivity**		.02		-.02

*Note*. SHS = Subjective Happiness Scale; NEO-FFI = NEO-Five Factor Inventory; BFPTSQ = Big Five Personality Trait Short Questionnaire; FFMQ = Five Facet Mindfulness Questionnaire. The BFPTSQ emotional stability scores indicate low neuroticism.

* *p* < .05.

** *p* < .01.

*** *p* < .001.

Table 4 shows the mean differences between the pre- and post-mindfulness training in personality, dispositional mindfulness and subjective well-being scores. Trait mindfulness (using gender, age and personality as covariates) improved significantly after the intervention (17.6%), with higher observing (15.0%) and describing (18.5%) facet scores. However, the other three mindfulness facets, personality dimensions and happiness were not affected by training. The results indicate that mindfulness practice improved dispositional mindfulness, especially attending and identifying experiences, but neither nonjudging, the main facet associated with happiness, nor happiness were modified.

**Table 4:** Means and standard deviations for personality, dispositional mindfulness and happiness, p values, Cohen’s d (Absolute Values) and partial Eta Squared associated with pre-intervention and post-intervention time points (n = 55).

	Pretest		Posttest				Partial η^2^
Scales	*M*	*SD*	*M*	*SD*	*d*	*F*	
***BFPTSQ*** ***(gender and age as covariates)***							
**Emotional stability**	19.00	8.56	19.69	8.48	.08	1.62	.030
**Extraversion**	26.05	7.69	26.20	6.76	.02	.17	.003
**Openness**	30.71	5.76	30.56	5.57	.03	.09	.002
**Agreeableness**	29.69	4.21	29.09	4.26	.14	1.69	.031
**Conscientiousness**	24.93	8.00	25.31	7.39	.05	.53	.010
***FFMQ*** ***(gender*, *age and the five factors of personality as covariates)***							
**Mindfulness (total score)**	46.29	8.13	48.95	7.23	.35	10.04*	.176
**Observing**	9.15	2.48	9.91	2.23	.32	8.31*	.150
**Describing**	9.49	3.05	10.31	2.62	.29	10.65*	.185
**Acting with awareness**	8.35	2.20	8.82	1.98	.22	2.81	.056
**Non judging**	10.67	2.52	11.02	2.78	.13	.98	.020
**Non reactivity**	8.64	2.39	8.89	2.00	.11	1.08	.023
***SHS*** **Subjective well-being**	19.49	4.35	20.04	3.89	.13	2.80	.056

*Note*. BFPTSQ = Big Five Personality Trait Short Questionnaire; FFMQ = Five Facet Mindfulness Questionnaire; SHS = Subjective Happiness Scale.

*p < .01.

## Discussion

The aim of this research was to examine the influence of the five factors of personality, dispositional mindfulness and mindfulness training on happiness. In relation to personality traits, and as expected, the correlations showed that subjective well-being was associated mainly with the extraversion and emotional stability broad domains. Considering that positive and (low) negative affects are two of the most important components of subjective well-being, and are respectively related to extraversion and emotional stability (low neuroticism), the findings confirmed the relevance of these personality traits in predicting happiness [26,27]. Dispositional mindfulness also correlated to happiness [7,13–15]. When controlling for personality in the multiple linear regression analyses, the dispositional mindfulness facets had a small incremental effect of 3% to 4% of unique variance in explaining the subjective well-being scores. Thus the present study indicates that the effects of dispositional mindfulness on predicting happiness were largely attributable to personality traits. These results were replicated in the two independent samples, even when using a different personality questionnaire in each group of participants. Nonetheless, only the nonjudging facet, a nonevaluative awareness of thoughts and feelings, still remained a significant predictor of subjective well-being in both samples when personality was accounted for. However, this association became nonsignificant in sample 2 when correction for multiple testing was applied. Previous studies have shown that a nonjudgmental attitude toward one’s thoughts and feelings to be the strongest predictor of well-being [50]. Contrarily to the study’s hypotheses, no other facet presented significant associations. One main explanation for the influence of the nonjudging facet on subjective well-being could be that being nonjudgmental (e.g., tending to think that one’s emotions and feelings are good or appropriate) contributes to control automatic thoughts, especially diminishing rumination, and to regulate negative affect and emotions such as worry and anger which, in turn, improve subjective well-being [17,24,50]. As already indicated, of all among the happiness components, (low) negative affect is one of the most important [27].

The results obtained for mindfulness intervention indicated, as predicted, that trait mindfulness (total score) increased, as former studies have found [18,35,36,51]. Nonetheless, other authors have not reported any increase after mindfulness training [52]. In the present research, only two facets improved, observing and describing, but it was hypothesized that the five facets would improve. Meta-analytic evidence [37] has indicated that mindfulness-related changes on observation, acting with awareness, nonjudging and nonreactivity are moderate, but the effect size for description is small. Thus the present results mainly replicated an improvement in the general mindfulness trait. One possible reason for these discrepancies could be duration of training. The adapted training herein used was reduced to six 2-hour sessions compared to the original MBSR, which consists of eight 2.5 hours sessions. Nonetheless, [18] used six weekly sessions of only 1.5 hours each and also found higher scores for the total mindfulness score in the treatment group than in the control group but, unlike the current results, they obtained significant improvements in all five facets. Moreover, [37] found that intervention length did not reliably influence the effects of training on the mindfulness facets. Another explanation could have something to do with the particular training employed in the present research. This was centered on metacognition exercises, which focused on observing thoughts at the present moment and being aware that the content to which the thought refers did not constitute a perception of the present. This characteristic of this modified MBSR intervention could explain why only observing and describing improved in the posttest, and not the other facets, as found in other studies with nonclinical samples (e.g., [35]).

In the present study, mindfulness training was not associated with subjective well-being, which falls in line with the results of the systematic review and meta-analysis by [51], who found little evidence for the effect of meditation programs on positive mood. Lack of support in the present research for the beneficial effect of mindfulness training on subjective well-being deserves further analysis. Previous findings seem to indicate that training leads to enhanced well-being, according to the meta-analysis results in clinical [53] and nonclinical [22] settings, and also in pioneering studies conducted in the workplace [9,35,36]. Furthermore, [37] found that beneficial intervention outcomes were associated moderately with changes in dispositional mindfulness facets, except for observation. These authors suggested that the facets expected to be trained could influence health-related changes. Accordingly, those interventions that focused on increasing well-being should improve nonjudging/acceptance [50]. The training program herein used, which centered especially on developing thought observation, improved observing and describing, but not the other facets of dispositional mindfulness or happiness. The intervention results of the present study do not demonstrate that mindfulness is related to subjective well-being through practicing nonjudging. Taking into account the results of the regression analyses, it seems reasonable to recommend that training programs should focus on cultivating a nonjudgmental/acceptance attitude toward one’s thoughts and feelings in order to increase happiness.

The present study has its limitations. In the first place, its cross-sectional design did not allow to establish the causal link among personality traits, mindfulness facets and happiness. Future studies could explore the mediating role of mindfulness between personality and subjective well-being with a prospective longitudinal design. Moreover, most of the participants were well-educated females, so replication studies not only have to use other designs, but also divergent samples. It would also be worth assessing the participants’ previous experience in mindfulness, meditation, or in other related areas such as yoga. Second, the mindfulness intervention was carried out in a nonclinical sample and there was no control group for comparison. Future research could also include clinical samples, which would benefit more from a mindfulness treatment in increasing the patients’ level of happiness. Third, the study only involved self-reports, and mindfulness was assessed using a short FFMQ version with lower internal consistencies than the full scale, although both seemed comparable [44]. The personality questionnaires differed in each sample, but this fact did not seem to affect the results about personality traits, which were very similar in the two independent samples. Using other data collection sources, such as peer-ratings and observations, might enrich the conclusions that could be drawn about the role of a nonevaluative awareness of thoughts and feelings in happiness.

Despite these limitations, and according to the findings in the present study, one conclusion could be that a nonjudgmental/acceptance attitude toward one’s inner thoughts, feelings and sensations contributed to happiness, but the effect was small, even when personality was taken into account. In relation to mindfulness training, the results highlighted that a mindfulness training, which centered on metacognition and focused on observing thoughts, did not increase subjective well-being. The present results did not demonstrate that an intervention directed to reducing judgmental attitudes would increase happiness. However, one reasonable recommendation would be that training programs should target and improve nonjudging if they intend to increase subjective well-being, as this facet contributed to happiness above and beyond personality traits.

## Supporting information

S1 Fig Mindfulness training. Metacognition-based mindfulness and meditation program.
(PDF)
