# Supplementary material for: Personality and nonjudging make you happier: Contribution of the Five-Factor Model, mindfulness facets and a mindfulness intervention to subjective well-being
Source: PLoS One. 2020 Feb 4;15(2):e0228655. doi: 10.1371/journal.pone.0228655 (PMC6999907; doi:10.1371/journal.pone.0228655)
Supplement: S1 Fig — Metacognition-based mindfulness and meditation program. (PDF) [file pone.0228655.s001.pdf]

ResearchGate

See discussions, stats, and author profiles for this publication at: https://www.researchgate.net/publication/336749548

METACOGNITION-BASED MINDFULNESS AND MEDITATION PROGRAM (MCbM&MP)

Research Proposal · October 2019

DOI: 10.13140/RG.2.2.18407.19367

CITATIONS

0

1 author:

Daniel Pinazo

Universitat Jaume I

37 PUBLICATIONS **233** CITATIONS

SEE PROFILE

Some of the authors of this publication are also working on these related projects:

Project Mindfulness View project

Project Social Communication View project

All content following this page was uploaded by Daniel Pinazo on 23 October 2019.

The user has requested enhancement of the downloaded file.

METACOGNITION-BASED MINDFULNESS AND MEDITATION PROGRAM

(MCbM&MP)

Daniel Pinazo-Calatayud
Social Psychology
Universitat Jaume I
Castellón de la Plana
Spain

Session 1. Mindfulness and metacognition

Session 2. Mindfulness training. The importance of perceiving reactive attention

Session 3. The conviction of being right instigates reactive attention

Session 4. Acceptance and emotional emotional self-regulation

Session 5. Attention to how you interpret experiences. Let meanings go, let your experience be.

Session 6. Are you grateful for what you live?

SESSION 1.

Mindfulness and metacognition

20’. Information about mindfulness, judgment formation and emotional meanings in interaction with experiences.

15’ Meditation and mindfulness practice

10’ Information about how we fussion the thought or felt meaning with situational self-concept.

10’ Practice of thinking separation, self-differentiation between what is thought/felt and situational self-concept.

15’ Information about the importance of breathing.

15’ Breathing practice.

20’: Sitting silent meditation practice, observing thoughts and metathinking about their detachment from our focus.

15’ Comments and task assignment for next week. The session may continue with participants’ comments and until the task is clarified.

Main practice for the week

1. Prepare a focused routine in different contexts during the day

   - Use single focal points in two or three settings where you are on auto-pilot.

   - Observe the moments when you find yourself judging. Observe yourself when you blame your emotional reaction on other people. As soon as you notice how your thoughts focus on the context bring your attention back to how you feel. Observe what is happening in your mind detaching yourself from judgement.

   -Informal practice: routine activities (shower, brushing teeth, focused attention during eating…)

2. Practice 20 breaths twice a day in your workplace or where you spend most of your time.

3. Practice body scan for 30 minutes every day.

4. Practice silent meditation observing wandering thoughts as they drift through the mind for 10 minutes every day.

SESSION 2.

Mindfulness training. The importance of perceiving reactive attention

15’ Walking meditation

20’ Review of last week’s main practice

30’ Body scan

20’ Information about reactive attention vs conscious attention (mindfulness). Reactive attention to the most salient aspects of information processing.

20’ Practise. Being aware of internal and external events as phenomena, rather than as objects of a conceptually constructed world.

15’ Comments and task assignment for next week. The session may continue with participants’ comments and until the task is clarified.

Main practice for the week

1. Prepare a focused routine in different contexts during the day
   - Use single focal points in two or three settings where you are on auto
   - Observe the moments when you find yourself judging. Observe yourself when you blame your emotional reaction on other people. As soon as you notice how your thoughts focus on the context bring your attention back to how you feel. Observe what is happening in your mind detaching yourself from judgement.
   - Informal practice: routine activities (shower, brushing teeth, focused attention during eating…)

1. Practice 20 breaths twice a day in your workplace or where you spend most of your time.

2. Practice body scan for 30 minutes every day.

3. Practice silent meditation observing wandering thoughts as they drift through the mind for 10 minutes every day.

SESION 3.

The conviction of being right instigates reactive attention

20’ Raisin exercise

20’ Review of last week’s main practice

20’ Practice: breathing, focusing on the wandering mind. Bringing attention back to breathing.

25’ Information on focusing on the internal dialog and the sense of authenticity it displays. Give up being right and focus on the emerging silence.

20’ Practice silent meditation, observing wandering thoughts and letting them go. Anchor attention on the breath.

15’ Comments and proposal of practices for the week. The session may continue with participants’ comments and until the task is clarified

Main practice for the week

1. Prepare a focused routine in different contexts during the day

   -Capture moments when you think you are right. Notice its presence. Give up being right.

   -Observe the moments when you think you are right. Notice the effect of judgment, the urge to defend it, the urge to defend its rationale. Can you suspend the urge to be right?

   -Informal practice: routine activities (showering, teeth brushing, focused attention during eating…)

2. Practice. Breath focus. 10-15 minutes daily.

3. Alternate body scan exercises and silent mediation. Do one of these tasks everyday for 20 minutes.

SESION 4.

Acceptance and emotional emotional self-regulation

20’: Mindful walking exercise

20´ Review of last week’s main practice

20’ Information about emotional meanings and how they define relationships with people and with our life experiences. Acknowledgement, acceptance, impersonal perspective.

10’ Emotional acceptance practice. Awareness of emotion and its meaning in close relationships.

15’ Information about metathinking activity when emotion is activated.

20’ Silent meditation observing the wandering of thoughts and letting them go.

15’ Comments and practise assignments for the week. The session may continue with participants’ comments and until the task is clarified

Main practice for the week

1. Prepare a focused routine in different contexts during the day

   - Capture moments when you feel you reject what you feel or think. Notice its presence. Accept, detach yourself from the emotion of rejection.

   - Observe the moments you make an effort to change whatever it is you do not like. Watch the effect of this effort, the way it bolsters the feeling of rejection or complaint. Can you suspend complaint?

   -Informal practice: routine activities (showering, teeth brushing, focused attention during eating…)

2. Practice. Breath focus. 10-15 minutes daily.

3. Alternate body scan exercises and silent mediation. Do one of these tasks everyday for 20 minutes.

SESSION 5.

Attention to how you interpret experiences. Let meanings go, let your experience be.

30’ Body scan

15’ Review of last week’s main practice

20’ Information on patterns or ideas that support judgments governing reactions.

20’ Focus on the wandering mind. Attention exercises on interpreting own experiences.

20’ Practice silent meditation observing wandering thoughts and letting them go. Anchor attention on breath.

15’ Comments and practise assignments for the week. The session may continue with participants’ comments until the task is clarified

Main practice for the week

1. Prepare a focused routine in different contexts during the day

-Capture moments when you perceive you reject what you feel or think. Notice its presence. Observe the meaning that defends that rejection. Accept the emotion, detach yourself from the emotion of rejection and from the thought that justifies it.

- Observer the moments that are not “the way they are supposed to be”. Observe the effects of following that norm or enforcing it. Can you let be what is not supposed to be?

-Informal practice: routine activities (showering, teeth brushing, focused attention during eating…)

2. Practice. Breath focus. 10-15 minutes daily.

3. Alternate body scan exercises and silent mediation. Do one of these tasks everyday for 20 minutes.

SESSION 6.

Are you grateful for what you live?

20’ Mindful meditation

15’ Review of last week’s main practice

15’ Information on how we stop paying attention to those everyday aspects we take for granted.

15’ Practice of attention to love or gratefulness.

20’ ’ Practice silent meditation observing wandering thoughts and letting them go. Anchor attention on breath.

15’ Comments and practise assignments for the week. The session may continue with participants’ comments until the task is clarified

20’ General review. Everybody is invited to speak.

Main practice for the week

1. Prepare a focused routine in different contexts during the day

   - Capture moments when you can stay grateful for the little things in everyday life. Focus on what you are grateful for and pay attention on how gratefulness arises in you.

   - Observe the moments when you are not grateful. Observe the origin of the feeling of non being grateful. Can you stay grateful? What blocks it? Detach yourself from the justifying dialogue.

   - Informal practice: routine activities (showering, teeth brushing, focused attention during eating…)

2. Practice. Breath focus. 10-15 minutes daily.

3. Alternate body scan exercises and silent mediation. Do one of these tasks everyday for 20 minutes.

View publication stats
